# Does individualism bring happiness? Negative effects of individualism on interpersonal relationships and happiness

**DOI:** 10.3389/fpsyg.2014.00135

**Published:** 2014-03-05

**Authors:** Yuji Ogihara, Yukiko Uchida

**Affiliations:** ^1^Department of Cognitive Psychology in Education, Graduate School of Education, Kyoto UniversityKyoto, Japan; ^2^Kokoro Research Center, Kyoto UniversityKyoto, Japan

**Keywords:** individualism, subjective well-being, interpersonal relationships, culture, cultural change, globalization

## Abstract

We examined the negative effects of individualism in an East Asian culture. Although individualistic systems decrease interpersonal relationships through competition, individualistic values have prevailed in European American cultures. One reason is because individuals could overcome negativity by actively constructing interpersonal relationships. In contrast, people in East Asian cultures do not have such strategies to overcome the negative impact of individualistic systems, leading to decreased well-being. To test this hypothesis, we investigated the relationship between individualistic values, number of close friends, and subjective well-being (SWB). Study 1 indicated that individualistic values were negatively related with the number of close friends and SWB for Japanese college students but not for American college students. Moreover, Study 2 showed that even in an individualistic workplace in Japan, individualistic values were negatively related with the number of close friends and SWB. We discuss how cultural change toward increasing individualism might affect interpersonal relationships and well-being.

## INTRODUCTION

The literature on cultural values has discussed the sizable cross-cultural differences between personal and interpersonal social values, such as individualism/collectivism (Hofstede, 1980; Triandis, 1995), and independence/interdependence (Markus and Kitayama, 1991). Theories and evidence have repeatedly suggested that individualism or independence is more frequently observed in European American cultural contexts whereas collectivism or interdependence is more frequently observed in East Asian cultural contexts.

However, globalization – “a process by which cultures influence one another and become more alike through trade, immigration, and the exchange of information and ideas” (Arnett, 2002, p. 774) – has been a powerful and unstoppable force in recent decades (Chiu et al., 2011) and cross-national or cross-cultural distinctions may be getting smaller. Globalization enables greater mobility of people, objects, money, and information across countries. Especially since the 1980s, international trade by transnational companies and enterprises has been expanding, and the ongoing developments in improved transportation and information technologies have created a globalized world. Globalization is not only making societies more international, but also more Westernized or European-Americanized. Indeed, globalization is sometimes called Americanization or Westernization (e.g., Guillen, 2001) and lay people perceive globalization to be related to the Western cultural values (Yang et al., 2011). This means that European American culture is one of the most potent cultures in the world that has a strong influence on other cultures due to the political and economical strengths of Western cultures, which continue to export not only products, technologies, and economic systems but also values, ideas, and beliefs. As a result, there have been many cultural changes, especially in East Asian cultures, that have been affected by the spread of westernized cultural values, ideas, practices, and systems. In this research, we investigated how psychological tendencies might be affected by cultural changes, with a specific focus on the spread of individualism.

### INDIVIDUALISM IN THE EUROPEAN AMERICAN CULTURAL CONTEXT

Individualism – “a social pattern that consists of loosely linked individuals who view themselves as independent of collectives” (Triandis, 1995, p. 2) – is one of the most influential “global values” (Pilkington and Johnson, 2003). Importantly, individualism has long been fostered in European American cultural contexts. For instance, previous studies suggested that individualism is fostered over time by economic systems (i.e., the lifestyle of herders compared to those of farmers and fishermen; e.g., Uskul et al., 2008), the Protestant ethic (e.g., Weber, 1920; Quinn and Crocker, 1999), the philosophy of ancient Greece (e.g., Nisbett, 2003), the decreased prevalence of pathogens, and voluntary settlements (e.g., Kitayama et al., 2006).

Individualistic systems or environments are believed to have positive influences on individuals (e.g., Waterman, 1981). For example, individualistic systems enable individuals to act autonomously and choose freely (Triandis, 1995), with high social mobility such as being able to choose desirable persons to interact with (e.g., Schug et al., 2009), which tends to increase happiness (Inglehart et al., 2008; Fischer and Boer, 2011). Furthermore, people in individualistic cultures can have strong sense of self-efficacy (Kitayama et al., 2004).

However, such individualistic systems or environments can also have potentially negative effects. In particular, individualistic systems urge people to pursue personal achievement, which creates competition between individuals (Triandis, 1995). These systems can also result in high social mobility, which lead to high social anxiety (Oishi et al., 2013). In addition, the focused attention on personal achievements can bear a significant cost on interpersonal relationships (Park and Crocker, 2005).

Even though having costs, individualism brings benefits such as enjoying free choice and strong sense of self-efficacy. One strategy to buffer against the negative affects of individualistic systems is developing interpersonal skills, usually employed in European American cultures, contexts, including seeking new interpersonal relationships (Oishi et al., 2013), engaging in self-disclosure (Schug et al., 2010), explicitly seeking social support (Kim et al., 2006) or maintaining a high relational mobility by choosing desirable persons with whom to interact (Schug et al., 2009; Yuki and Schug, 2012). In short, in European American cultures, people are independent from each other (Markus and Kitayama, 1991, 2010) but still *actively* seek interpersonal relationships. Such interpersonal skills are probably acquired over an extended period through socialization, and allow people in these cultural contexts to enjoy interpersonal relationships while maintaining their independence.

### INDIVIDUALISM IN A JAPANESE CULTURAL CONTEXT

Through globalization, Japanese society has been influenced by European American cultures. This is especially true for the aspects of Japanese society that are adopting the individualistic systems imported from European American cultures. For example, the number of companies introducing pay-per-performance systems in Japan has increased (Institute of Labor Administration, 2004). Moreover, it has been argued that education that fosters children’s autonomy has recently been emphasized in schools (Doi, 2004). With the increase of individualistic environments in Japan, people have also become more individualistic in certain respects^1^. For instance, the average family size has decreased, the divorce rate has increased, and independence in child socialization has been increasingly prioritized (Hamamura, 2012).

However, it has been argued that individualism in Japan might be qualitatively different from the individualism in the European American cultural contexts (Kitayama, 2010). Individualism in these cultural contexts means being independent from others but still actively making social relationships. By contrast, to be independent and achieve “individualism,” the Japanese might feel the need to distance themselves from interdependent relationships. Indeed, connotations of individualism in Japan are more negative than are those in the U.S. Specifically, in the U.S. individualism is perceived to be unique or independent, while in Japan individualism is regarded as being selfish and feeling lonely (Ogihara et al., 2013a,b). Unlike in European American cultural contexts, relational mobility is relatively low in East Asian cultural contexts; that is, people tend to interact with others with whom they already have a connection (Yuki and Schug, 2012). Hence, the Japanese are more likely to commit to a long-term relationship rather than to seek new relationships (Yamagishi and Yamagishi, 1994). However, long-term, pre-existing interpersonal relationships can bind and restrict individuals because these relationships are often rule-based, not autonomy-based. Therefore, it might be necessary for Japanese individuals to cut off traditional relationships to be independent. Moreover, once these relationships are cut off, it is difficult for the Japanese to develop new relationships. Even under the motivation to be independent, the Japanese do not actively create new relationships because they are not equipped with appropriate strategies for making and constructing new social relationships, such as actively engaging in self-disclosure (Schug et al., 2010) or explicitly seeking social support (Kim et al., 2006). In European American cultures, individualism has been fostered over a long period, so people have adequate strategies which have been acquired through socialization. In contrast, Japan was not an individualistic culture and the exposure to individualization is comparatively recent. Therefore people in Japan might not have the strategies which are appropriate in an individualistic culture. As a result, under individualistic systems, Japanese tend to cut off interpersonal relationships but do not actively build new close interpersonal relationships. Thus becoming more individualistic might decrease Japanese happiness because interpersonal relations are an important source of happiness in Japan (e.g., Uchida et al., 2008).

### PRESENT STUDY

We examined the relationship between individualistic values, subjective well-being (SWB), and number of close relationships in Japan and the U.S. Study 1 tested the hypothesis that individualistic values would be associated with a decrease in the number of close friends and SWB in Japan, but not to close friends and SWB in the U.S. Furthermore, to examine the effect of individualistic values and structural systems, Study 2 tested if decreases in the number of close relationships and SWB would be found in a sample of adults working in an individualistic environment in Japan. We predicted that even in a workplace that has individualistic systems and requires individualistic values, individualistic values would be negatively related to the number of close friends and SWB for Japanese workers.

## STUDY 1

Study 1 investigated whether individualistic values would have different effects on SWB across cultures. We predicted that (1) an individualistic orientation would decrease SWB in Japan, but not in the U.S., and (2) fewer close relationships would mediate the negative effect of individualistic values on SWB in Japan, whereas individualistic values would not be related to the number of close relationships in the U.S.

## METHOD

### PARTICIPANTS AND PROCEDURE

One hundred and fourteen undergraduate students at Kyoto University in Japan (62 male, 52 female; *M*_age_ = 19.5, *SD*_age_ = 1.77) and 62 undergraduate students at University of Wisconsin-Madison in the U.S. (29 male, 33 female; *M*_age_ = 19.3, *SD*_age_ = 1.17; 60 White and 2 Hispanic born in the U.S.) participated in this study.

### MEASURES

#### Individualistic and collectivistic orientations

We used the revised version of the Contingencies of Self-Worth Scale^2^ (Crocker et al., 2003; Uchida, 2008). This scale assesses 11 domains of self-worth (e.g., academic competence, relationship harmony). Factor analysis in each culture indicated that 9 of the 11 domains fell into two factors, namely, individualistic orientation (e.g., academic competence, and competition; α_Japan_ = 0.92, α_US_ = 0.89) and collectivistic orientation (e.g., relationship harmony and other’s support; α_Japan_ = 0.89, α_US_ = 0.81).The other two domains (support of family and virtue) were dropped from the analysis because of the low factor scores. A sample item in individualistic orientation is “Doing better than others gives me a sense of self-respect,” and an example of the items in collectivistic orientation is “I can’t respect myself if I break relationship harmony within my group.” Participants reported the degree to which each statement applied to them (1 = *strongly disagree*, 7 = *strongly agree*).

#### Subjective well-being

Participants completed four scales to assess their SWB. First, we measured life satisfaction by using the Satisfaction With Life Scale (Diener et al., 1985; five items; e.g., “In most ways, my life is close to my ideal”). Second, the Interdependent Happiness Scale (Hitokoto et al., 2009; 32 items) was used to measure individual differences in interdependent happiness gained by maintaining harmony with significant others (e.g., “I believe that I and those around me are happy”). Participants answered these two measures on a 7-point scale (1 = *strongly disagree*, 7 = *strongly agree*). Third, we assessed positive and negative affect. Positive affect was measured with 11 items (e.g., happy, satisfied) and negative affect was measured with 15 items (e.g., depressed, sad). Fourth, we measured somatic symptoms (11 symptoms; e.g., headache and stiff joints). Participants reported how frequently (1 = *never*, 5 = *very often*) they experienced each of these affective states and somatic symptoms. All of these items have been successfully used in a survey of Midlife Development in the U.S. (MIDUS; Brim et al., 2004). All of these scales had satisfactory internal consistency (0.72 < α s < 0.90).

#### Number of close friends

The number of close friends was measured using a sociogram (Kitayama et al., 2009). The sociogram is a simple diagram that shows an individual’s interpersonal relationships. Participants were asked to draw circles representing themselves and their friends on a paper and to connect related persons with lines within 10 min. After this was done, they were asked to identify the friends with whom they feel comfortable. The “close friend” variable was defined as the number of people with whom the participants felt comfortable.

## RESULTS

### ORIENTATIONS IN JAPAN AND THE U.S.

**FIGURE 1:**
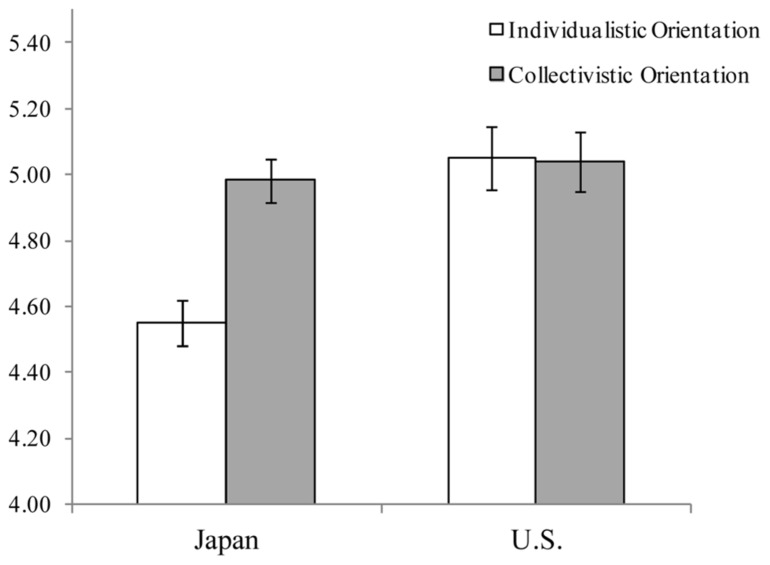
**Raw scores of individualistic and collectivistic orientations in Japan and the U.S. (Study 1).** Bars represent the standard error.

The average raw scores of individualistic and collectivistic orientations are shown in **Figure 1**. We conducted a 2-way (orientation and culture) ANOVA and found main effects of both orientation [*F*(1,174) = 11.68, *p *< 0.001, $\eta_{p}^{2}$ = 0.06] and culture [*F*(1,174) = 8.97, *p *< 0.01, $\eta_{p}^{2}$ = 0.05]. In addition, the interaction between orientation and culture was significant [*F*(1,174) = 12.57, *p *< 0.001, $\eta_{p}^{2}$ = 0.07]. The individualistic orientation score was significantly higher for the U.S. participants than for Japanese participants [*F*(1,173) = 17.80, *p *< 0.001], whereas the collectivistic orientation score was not significantly different across cultures [*F*(1,173) = 0.26, *p *= 0.61].

### EFFECTS OF ORIENTATIONS ON SWB

**FIGURE 2:**
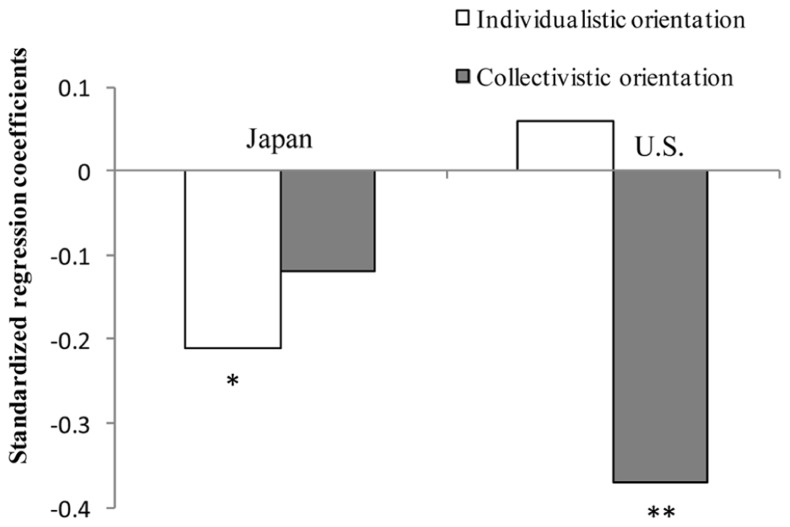
**Standardized regression coefficients predicting subjective well-being in Japan and the U.S. (Study 1).** **p < 0.01, *p < 0.05.

Due to generally consistent results across the four SWB measures, a single SWB index was developed using a principle component analysis (**Table 1**). Multiple regression analysis was conducted to examine how individualistic and collectivistic orientations affected SWB in each culture^3^. In Japan, an individualistic orientation negatively affected SWB, whereas a collectivistic orientation did not affect SWB (**Figure 2**). In contrast, in the U.S., a collectivistic orientation negatively affected SWB, and an individualistic orientation did not affect SWB.

**Table 1:** Principle component scores of SWB index in Japan and the U.S. (Study 1).

	Japan	U.S.
Interdependent happiness	0.89	0.88
Negative affect	-0.78	-0.82
Positive affect	0.78	0.80
Life satisfaction	0.73	0.86
Somatic symptoms	-0.70	-0.70

### MEDIATION EFFECT OF NUMBER OF CLOSE FRIENDS

**FIGURE 3:**
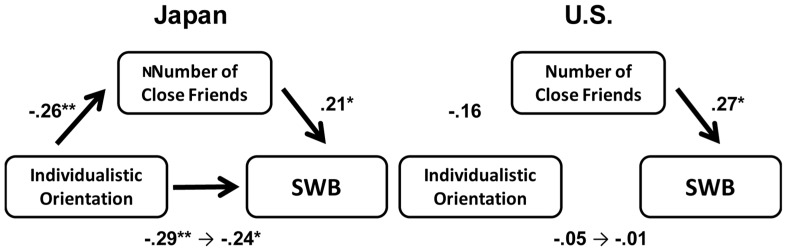
**Mediation effect of the number of close friends between individualistic orientation and SWB in Japan and the U.S. (Study 1).** Path coefficients on the left side of the arrows from individualistic orientation to SWB indicate standardized regression coefficients when individualistic orientation is a single independent variable. Those on the right side of the arrow indicate standardized regression coefficients when both individualistic orientation and the number of close friends are independent variables. Gender and age were controlled. **p < 0.01, *p < 0.05.

We conducted a mediation analysis to test whether the number of close friends mediated the effect of an individualistic orientation on SWB. The distributions of the numbers of close friends were positively skewed. Thus, we transformed the values by computing their common logarithm (plus 1), which produced an approximately normal distribution (using the same analysis proposed by Kirkpatrick et al., 2002). In Japan, an individualistic orientation was associated with fewer close friends. Moreover, the number of close friends positively predicted SWB, even after orientation was controlled. Therefore, in Japan the number of close friends mediated the effect of an individualistic orientation on SWB (**Figure 3**). Furthermore, a Sobel test (Sobel, 1982) indicated that the mediating effect of the number of close friends was marginally significant (*z* = -1.75, *p* = 0.08). In contrast, we did not find such a relationship in the U.S.

## DISCUSSION

As predicted, in Japan an individualistic orientation was negatively related to SWB, but not in the U.S. In addition, in Japan, the number of close friends mediated the negative effect of an individualistic orientation on SWB. This suggests that if people in Japan try to be independent and achieve individualism, they will have difficulty forming and maintaining close friendships. Interestingly, however, there was no relationship between an individualistic orientation and the number of close friends in the U.S. Therefore, we concluded that the effect of individualistic values differs between Japan and the U.S. Specifically, individualistic values in Japan were associated with a deterioration in close relationships and a decrease in SWB, whereas individualistic values in the U.S. did not have a negative effect on close relationships and SWB.

## STUDY 2

Study 1 revealed that in the U.S. an individualistic orientation did not influence SWB and interpersonal relationships, whereas in Japan individualistic orientation was negatively associated with SWB and the number of close friends. However, these results might be questioned if the negative impact of individualistic orientation in Japan was due to the conflict between individualistic orientation in personal level and the collectivistic social structure.

Therefore, in Study 2, we chose a sample of women working in an individualistic-orientated workplace to examine whether negative impact of individualism found in Study 1 could be generalized to an individualistic-oriented working environment in Japan. We examined whether people with individualistic orientations working in an individualistic social structure would exhibit the same negative effects of individualism as exhibited by the Japanese participants in Study 1.

## METHOD

### PARTICIPANTS AND PROCEDURE

Thirty-four women (*M*_age_ = 26.6, *SD*_age_ = 6.19, 22–51 years old) who worked for a large insurance company in Japan participated in the study. The data of two participants who reported they had lived abroad for more than 5 years were excluded. In this insurance company, performances and achievement-oriented goals are explicitly displayed on the wall (e.g., how many contracts each individual secured in the past 1 month); such displays are perceived as competitive. Participants answered the same questionnaire used in Study 1; all scales had satisfactory internal consistency (α _individualistic orientation_ = 0.90, α _collectivistic orientation_ = 0.87, 0.79 < α _SWB scales_ < 0.95).

## RESULTS

### ORIENTATIONS IN ADULT SAMPLES AND STUDENT SAMPLES

**FIGURE 4:**
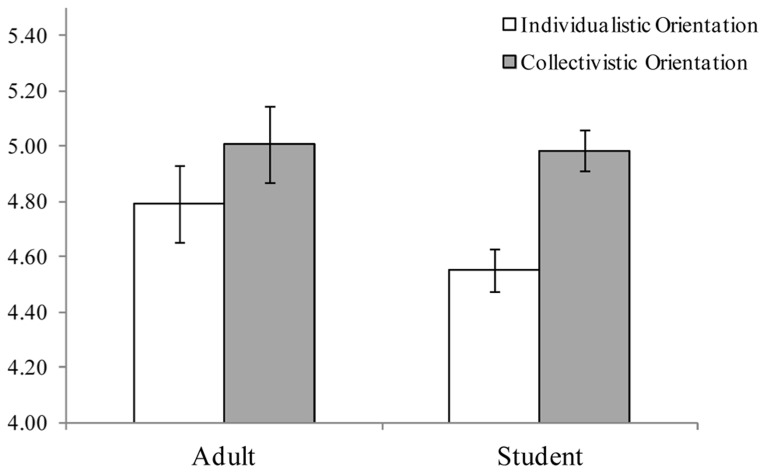
**Raw scores of individualistic and collectivistic orientations in adult samples and student samples in Japan.** Bars represent the standard error.

Raw scores for individualistic and collectivistic orientations in the adult and student samples are shown in **Figure 4**. We conducted a two-way (orientation and group) ANOVA and found a main effect of orientation [*F*(1,144) = 17.97, *p *< 0.001, $\eta_{p}^{2}$ = 0.11]. In contrast, the main effect of group was not significant [*F*(1,144) = 0.89, *p *= 0.35, $\eta_{p}^{2}$ = 0.01] nor was the interaction between orientation and group [*F*(1,144) = 2.07, *p *= 0.15, $\eta_{p}^{2}$ = 0.01].

### RELATIONSHIPS BETWEEN INDIVIDUALISTIC ORIENTATION, THE NUMBER OF CLOSE FRIENDS, AND SWB

**FIGURE 5:**
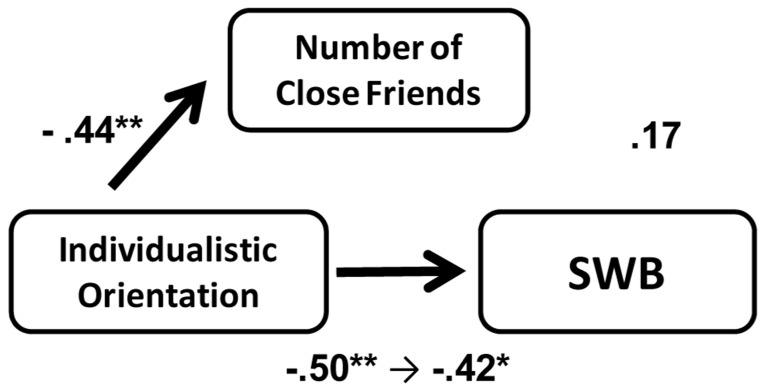
**Effect of individualistic orientation on SWB and number of close friends in Japanese individualistic-oriented environment (Study 2).** Path coefficients on the left side of the arrow from individualistic orientation to SWB indicate standardized regression coefficients when individualistic orientation is a single independent variable. Those on the right side of the arrow indicate standardized regression coefficients when both individualistic orientation and the number of close friends are independent variables. Age was controlled. **p < 0.01, *p < 0.05.

The results were consistent with Study 1; an individualistic orientation was negatively associated with both SWB and the number of close friends (**Figure 5**). Although the number of close friends positively predicted SWB (β = 0.41, *p* < 0.05), when the effect of individualistic orientation was controlled, the effect of close friends on SWB became weak (β = 0.17, *p* = 0.42). However, importantly, individualistic orientation had a negative relationship with both SWB and the number of close friends.

## DISCUSSION

We found that an individualistic orientation was negatively associated with the number of close friends and SWB even for women working in an individualistic-oriented workplace. This result was the same as in the college sample in Study 1; however, we did not find a mediating relationship of close friends. One explanation for the lack of relationships between the number of close friends and SWB might lie in the sample; specifically, for the women working in an individualistic-oriented workplace, the achievement of individualistic goals required in the workplace may be more important to SWB than positive relationships with others. The result suggested, however, that even in an achievement-oriented environment in Japan, achievement-oriented individuals feel lower SWB and have fewer close friends. Thus, it is indicated that Japanese with individualistic orientations have fewer close friends and feel lower SWB.

## GENERAL DISCUSSION

We examined the effect of individualistic values on SWB in two studies. Study 1 demonstrated that an individualistic orientation was not associated with decreased SWB in the U.S., whereas an individualistic orientation was associated with fewer close friends and lower SWB in Japan. Furthermore, Study 2 showed that an individualistic orientation was also associated with a decrease in the number of close friends and SWB for adult women working in an individualistic-oriented workplace. These results suggest the negative effect of an individualistic orientation in Japan might be due to the lack of a “buffer” against the negative impact of individualism within individuals (i.e., developing new interpersonal relationships).

### NEGATIVE EFFECTS OF INDIVIDUALISM IN EAST ASIAN CULTURAL CONTEXT

The results showed that an individualistic orientation dampened close interpersonal relationships and SWB in Japan, suggesting that individualism has a negative effect in East Asian cultural contexts. Although this study examined individualistic values in Japan, the results may generalize to other East Asian countries (e.g., China and South Korea) since a number of studies have shown that East Asian countries have traditionally interdependent or collectivistic cultural norms (e.g., Choi et al., 1997).

Recently, Japanese systems and environments are becoming more individualistic, but people in Japan may not respond well to these new systems. Why is there a gap between the environments and individual’s psychological tendencies such as personal values? We expect that this gap between environments and individuals’ psychological tendencies is due to unsuccessful attempts to adapt to new environments (i.e., individualistic systems in Japan) without effective behavioral strategies. To be more specific, when individuals in Japan try to be independent, they may cut off their existing relationships, and may not possess strategies to actively build new interpersonal relationships, unlike independent individuals in the European American cultural contexts where actively building new interpersonal relationships is common (e.g., Schug et al., 2010; Oishi et al., 2013).

Individualism in the European American cultural context is based on shared values and the notion that individuals are “independent from each other,” but still “connected with each other.” In European American cultural contexts, this sense of values and behavioral strategies are fostered through long historical periods (e.g., Nisbett, 2003;). In contrast, it is only recently that individualistic systems or environments have been drastically imported to East Asian cultural context. Therefore, these environments are comparatively new, and the Japanese imported individualism might consist only of parts of Western individualism. For example, even though Japanese companies or schools use personal achievement systems, these systems are not backed by the personal values that govern these systems in European American cultural context, such as active interpersonal strategies, religious ideas, or high self-efficacy. Therefore, it remains difficult for East Asians to buffer the negative effects of individualistic systems or environment.

The novelty of this study is that it separetes values and interpersonal strategies, especially as seen in the results of Study 2. Although the Japanese might espouse individualistic values, they might not be able to achieve the same positive consequences of individualism that are observed in European American cultural contexts because they are not equipped with the strategies necessary to buffer the negative interpersonal effects of individualism. Although some researchers have theoretically distinguished social structure from values (e.g., Dimaggio and Markus, 2010), no research has treated values and strategies as separate constructs. Our findings reveal the simplicity of structure versus values distinction by highlighting the importance of the strategies that people use to act out their values. Transplanting both the structure and the values of one culture into another might not work if individuals do not have the strategies to adaptively act out their values in the given structural setting. It is especially important to consider that the recent rapid changes of social structures and values may not be accompanied by behavioral strategies, which take longer to develop through many social training. Although cross-cultural differences in social behavior are well established, much less is known about how cultural change influences individuals. Further research should be conducted to examine the effect of cultural changes on human psychology and behavior.

### LIMITATIONS AND FUTURE RESEARCH

We explained that in individualistic cultures, such as American culture, people acquire strategies to deal with the negative interpersonal consequences of individualism through long-term socialization. By contrast, in Japan, it is comparatively recent that society has become individualistic and, even when people have individualistic values, they are not well equipped with appropriate strategies to buffer the negative effect of individualism. However, another interpretation is possible; having an individualistic personal orientation might conflict with Japan’s traditional collectivistic values in Japan. In order to conclude which is the better explanation, it may be important to examine the buffering effect against individualism, such as by collecting data from people who appear to have better buffers against the negative effects of competitive working environments.

We examined the relationships between an individualistic orientation, the number of close friends, and SWB in Japan and the U.S., but we did not test the causal relationships directly (i.e., an individualistic orientation decreased the number of close friends and, as a result, SWB decreased). It is possible that having a small number of friends leading to individualism and lower SWB. Investigating the causal associations between an individualistic orientation, social relationships and SWB by longitudinal survey study or using the accumulated archive data could be a focus of future research.

Although not part of our hypothesis, we found that a collectivistic orientation was negatively associated with SWB in the U.S. We could not find evidence to suggest reasons for this decrease (in contrast to finding the connection to the number of close friends in Japan). This point should be explored further.

### IMPLICATIONS FOR GLOBALIZATION

Our findings provide insight on the effect of globalization in the case of not only individuals but also nations. Of course, globalization has many benefits for individuals and nations. We can get together frequently due to transportation innovations, products that are made in distant locations are readily available, and we can learn almost anything via information technology. However, there are also some negative effects. Globalization may cause maladaptive responses and deviations from mainstream culture, especially outside European American cultures (e.g., Asian or African cultures). It may be that such deviations from the cultural mainstream are linked to current social issues, such as social withdrawal (e.g., *hikikomori* in Japan who isolate themselves into their own bedrooms from 6 months to decades at a time without interacting with others, sometimes even with their own families; Norasakkunkit and Uchida, 2011). In addition, values and systems that are adaptive in European American cultures may not necessarily be adaptive in other cultures (e.g., Oishi, 2012). Thus, it might be necessary for each nation to select which values and systems to endorse, or introduce new ones in ways that are compatible with their culture. For instance, Bhutan, a small Asian country located between China and India, has gained much attention because of its distinctive policy against globalization (Center for Bhutan Studies, 2012). The Bhutan government explicitly states that not only the physical happiness (Gross National Product) but also the psychological happiness (Gross National Happiness) of citizens are key policies of the country.

In a more globalized world, culture matters more than ever before. Therefore, the effect of globalization (in particular, the effects of individualism) on individuals and nations should be examined from a cultural perspective in more detail in the future.

## AUTHOR CONTRIBUTIONS

Yuji Ogihara and Yukiko Uchida jointly carried out all the empirical work and wrote the manuscript.

## Conflict of Interest Statement

The authors declare that the research was conducted in the absence of any commercial or financial relationships that could be construed as a potential conflict of interest.

## Footnotes

1 It should be noted that there were some indices which did not show that Japanese have become more individualistic (Hamamura, 2012). However, all the indices that did not show the increase in individualistic tendency were self-report items of beliefs and values. In contrast, behavioral measures (i.e., average family size, divorce rate, and proportion of people living in urban areas) documented an increase of individualism. Previous research suggested that self-report items have some problems. For example, people tend to compare themselves with individuals in their culture rather than those in another culture (the reference-group effect; Heine etal., 2002). Therefore, even though one may have become more individualistic, they might not realize their increase of individualism due to a reference-group effect.

2 We chose this scale because it can successfully measure individualistic and collectivistic orientation with relative implicitness. If we used a more direct measure, that included items such as “I like doing better than others” or “I do not like breaking relationship harmony within my group,” participants might not report their actual orientation or attitudes because of social desirability.

3 We conducted a multiple regression analysis with independent variables of individualistic orientation, collectivistic orientation, culture, the interaction term between culture and individualistic orientation, the interaction term between culture and collectivistic orientation, age, and gender, with SWB as the dependent variable. We found a significant effect of individualistic orientation (β = -0.22, *p* < 0.05), but the interaction term between culture and an individualistic orientation was only close to be marginal (β = 0.16, *p* = 0.105).
